# Stroller running: Energetic and kinematic changes across pushing methods

**DOI:** 10.1371/journal.pone.0180575

**Published:** 2017-07-03

**Authors:** Ryan S. Alcantara, Cara M. Wall-Scheffler

**Affiliations:** 1 Department of Biology, Seattle Pacific University, Seattle, Washington, United States of America; 2 Department of Anthropology, University of Washington, Seattle, Washington, United States of America; Norwegian University of Science and Technology, NORWAY

## Abstract

**Objective:**

Running with a stroller provides an opportunity for parents to exercise near their child and counteract health declines experienced during early parenthood. Understanding biomechanical and physiological changes that occur when stroller running is needed to evaluate its health impact, yet the effects of stroller running have not been clearly presented. Here, three commonly used stroller pushing methods were investigated to detect potential changes in energetic cost and lower-limb kinematics.

**Methods:**

Sixteen individuals (M/F: 10/6) ran at self-selected speeds for 800m under three stroller conditions (2-Hands, 1-Hand, and Push/Chase) and an independent running control.

**Results:**

A significant decrease in speed (p = 0.001) and stride length (p<0.001) was observed between the control and stroller conditions, however no significant change in energetic cost (p = 0.080) or heart rate (p = 0.393) was observed. Additionally, pushing method had a significant effect on speed (p = 0.001) and stride length (p<0.001).

**Conclusions:**

These findings suggest that pushing technique influences stroller running speed and kinematics. These findings suggest specific fitness effects may be achieved through the implementation of different pushing methods.

## Introduction

Running is a popular form of physical activity, with over 17 million individuals finishing a U.S running event in 2015 (2016 Road Running Report). Jogging strollers were first developed in the 1980’s and have since become popular amongst runners with young children. As a physical activity that can be performed near the child, stroller running appeals to parents looking to counteract health declines often experienced by postpartum men and women [1–3]. Despite the popularity of stroller running (SR) in the running community, there has been limited research on its effects on physiological and biomechanical variables [4–8], making it difficult to assess how SR might impact a runner’s gait or energy expenditure compared to running independently.

Given the complexity of pushing mass during locomotion [9–10], it is not completely clear what might happen biomechanically during the gait cycle during SR. Additionally, there seem to be many options as to how a runner might integrate a stroller into his or her routine, possibly trying a variety of pushing techniques to maintain some particular aspect of gait that is either more comfortable or minimizes energy expenditure. Methodological differences between previous studies, particularly regarding condition intensity (Table 1) makes an assessment of SR additionally challenging. For example, a series of authors had participants run at racing paces or high percentages of VO_2_ Max, which are potentially not the paces that many people choosing to run with strollers recreationally will experience [5–6]. Due to these methodological differences and possible inconsistencies with how people perform SR, their results are not easily applied to the general SR population. Information regarding the prevalence of different stroller pushing methods within the general SR population would prove valuable when developing a study investigating the effects of SR.

**Table 1:** Review of prior studies investigating stroller running.

Study #	Authors	Participants	Distance/ Duration	Surface	Speed	Pushing Method	Stroller	Load	Results
**1**	*Brown et al*. *(2008*)	8♀, collegiate cross-country runners	2.4 km	Sidewalk	"racing or fast training" pace	-	Gerry Zoomer	9 kg	HR ↑, VO_2_ ↑, SL ↓, No Change: RPE, Speed
**2**	*Smith et al*. *(2005)*	5♂, 5♀, recruited from races	30 min	Outdoor track	75% of VO_2_ MAX	No Instruction	D’lux BOB Sports Utility Stroller	13.6 kg	HR ↑, Vent ↑, RPE ↑, No Change: VO_2_, SL, Speed
**3**	*O'Sullivan et al*. *(2015)*	5♂, 10♀, recently run 5k, no stroller exp. within 12 months	16 meter (x5)	Indoor Runway	Self-selected speed	2-Hands	Out n About Nipper Single	10 kg	Ant. trunk lean ↑, Ant. pelvic tilt ↑, Hip ext.↓, Trunk rot. ↓, No Change: Speed, SL
**4a**	*Gregory et al*. *(2011)*	6♂, 9♀, previous stroller experience	1.61 km	Indoor track	2.68 m*s^-1^	-	Baby Jogger Performance Series	11.36kg (I)	Vent ↑, No Change: VO_2_, HR, Cr, RPE
								22.72 kg (II)	RPE ↑, HR ↑ (II > I), No change: VO_2_, HR, Cr
			1.61 km	Indoor track	Self-selected Speed	-	Baby Jogger Performance Series	11.36kg	Vent ↑, No change: VO_2_, HR, Cr, RPE
								22.72 kg	No change: Vent, VO_2_, HR, Cr, REP
**4b**	*Gregory et al*. *(2011)*	3♂, 9♀, previous stroller experience	1.61km (on .80 km path)	Paved outdoor path	2.68 m*s^-1^	-	Baby Jogger Performance Series	11.36kg	VO_2_ ↑, Cr ↑, No change: HR, RPE, Vent
								22.72 kg	VO_2_ ↑, Cr ↑, RPE ↑, No change: HR, Vent
			1.61km (on .80 km path)	Paved outdoor path	Self-selected speed	-	Baby Jogger Performance Series	11.36kg	VO_2_ ↑, Cr ↑, RPE ↑ No change: HR, Vent
								22.72 kg	VO2 ↑, Cr ↑, RPE ↑, Vent ↑, No change: HR

Cr = Running Cost per unit time, HR = Heart Rate, RPE = Rate of Perceived Exertion, SL = Stride Length, Speed = Running Speed, Vent = Ventilation, VO_2_ = Oxygen Consumption

Even when comparing previous studies, there are conflicting results regarding the changes in physiological variables during SR (Table 1). Brown et al. [5] observed similar effects between VO_2_ and HR when comparing independent running to SR, yet Smith et al. [6] found that SR had significant effects on HR but no effect on VO_2_; however, Gregory et al. [7] observed increases in VO_2_, energetic cost, and RPE, yet no significant change in HR. These results are not only inconsistent, but also perplexing considering the established relationship between VO_2_ and HR when running [11–12]. Gregory et al. [7] observed differences in ventilation, HR, and RPE during SR on an indoor track, but observed no difference in VO_2_. The lack of a change in VO_2_ may be because participants received instruction during their run to maintain their pace, causing their values to be slightly elevated at all conditions due to interactions with researchers. These studies address physiological factors like HR, energetic cost, and oxygen consumption, which could prove valuable for individuals looking to make educated decisions regarding their physical activity but the methodological inconsistencies prevent an accurate understanding of the effects of SR.

Additionally, few studies have investigated biomechanical responses specific to SR, and there is no current consensus on how running speed and stride length are affected. Most recently, O’Sullivan et al. [8] and Smith et al. [6] observed no difference in stride length or running speed during SR compared to independent running, yet prior work by Brown et al. [5] noted changes in stride length. Differences in study design may have contributed to these confounding speed, stride length, and stride frequency results (Table 1).

Moreover, previous studies have not investigated the effects of different stroller pushing methods on physiological or biomechanical variables. Given that there are multiple ways for individuals to push a stroller while running, pushing method should be considered when making conclusions regarding the effects of SR on physiological variables. The interaction between the upper limbs and stroller when running not only changes upper body kinematics, but possibly running economy. Changes to arm swing patterns have been found to increase energetic cost, and different stroller pushing methods require different upper limb orientations [13].

Consequently, the purpose of this study was to investigate the energetic and kinematic effects of 1) stroller running compared to running independently, and 2) commonly used pushing methods used during stroller running in recreational runners. The methodology of the present study allowed for observations to be made under conditions similar to what was observed in recreational stroller runners in an urban environment. It was hypothesized that different pushing methods will result in changes in running speed, lower-limb kinematics, and energetic cost.

## Methods

### Prevalence

SR conditions were determined following observations of 290 individuals (151 men, 139 women) running with strollers in a public recreational area. Of those observed, approximately 78% chose a single method for the 400 meter duration of observation; the other 22% alternated between pushing methods. The three pushing methods observed in either case were classified as 1-Hand (42%), 2-Hands (51%), and Push/Chase (7%) (Push/Chase was defined as alternating between pushing the stroller ahead of the runner with both hands and running independently behind the stroller). As all runners observed used these three methods, each was compared against running independently in order to determine how pushing method might influence SR energetic cost and kinematics.

### Participants

Running economy and kinematics were measured in 16 participants (10 men, 6 women), who had no previous SR experience. Participants with no SR experience were selected to prevent bias towards a single pushing method. Mean subject age was 22.8 ± 3.3 (SD); mean body mass was 68.2 kg ± 11.9 (SD); and all participants ran at least 3 times a week for a minimum duration of 30 min. This study adhered to ethical research standards and was approved by the Seattle Pacific University Institutional Review Board. All participants signed consent forms prior to participation. This study excluded participants who had experienced an orthopedic injury within six months prior to study participation.

### Materials and protocol

Each participant ran 800 meters on an outdoor 400 meter track for three stroller conditions (1-Hand, 2-Hands, and Push/Chase) and one control which required running independently. No other people were on the track for the duration of the trial. Previous research has shown that 800m of comfortable jogging (see below for ‘comfortable’ definition) is the appropriate amount of time to have runners reach steady state values for each condition, and also not have a fatigue effect influence the later conditions [14–15]. All stroller conditions were performed with a Single Sport Stroller (phill&teds, Wellington, NZ) loaded with a 16kg weighted infant model in order to simulate the presence of a 3 year-old child [16]. There was an opportunity for participants to familiarize themselves with the stroller and adjust handle height prior to being fitted with the respirometer and HR monitor. All conditions were randomized for each subject and completed consecutively. After the completion of all the conditions, participants were asked to select which SR condition was the ‘most comfortable’ for them.

To measure running economy, subjects ran at self-selected speeds (mean speed = 3.1 ms^-1^ ± 0.5) while wearing a mobile respirometer (Oxycon Mobile, CA, USA) to collect breath-by-breath metabolic data [17]. The mobile respirometer was calibrated on-site immediately prior to each data collection and there were no significant differences in environmental conditions for each trial. Heart rate (HR) was also collected using a chest-strap HR monitor (Polar Electro, Finland). Participants consumed no caffeine or alcohol within 24 hours or any food within 4 hours of the trial to ensure accurate metabolic readings. Participants initially sat quietly for 5 minutes in order to obtain baseline metabolic rate and were then instructed to run at a speed they were comfortable maintaining for 60 minutes across all four conditions. Participants received the same verbal description of the requested pace at the beginning of their trial and this was repeated at the beginning of each condition. Participants were given a minimum of 4 minutes of rest between conditions to ensure metabolic rate returned to pre-exercise values; metabolic rate was monitored during the rest so that values did reach the initial resting values between each condition. Mean condition duration for each 800m run was 4.5 min ± 0.8 (SD); the metabolic data used for analysis was collected from the final 400 meters of the condition to ensure participants had reached a steady metabolic rate. Metabolic rate was converted from VO_2_ and VCO_2_ into Joules following Weir’s standard equation [18].

Stride frequency and running speed were measured with a stopwatch. Speed (ms^-1^) was measured every 100 meters and stride frequency (strides*s^-1^) was measured every 200 meters (during straightaways). Speed and stride frequency were averaged for the final 400 meters of each condition, and stride length (m/stride) was calculated from speed and stride frequency.

### Data analysis

A repeated-measures ANOVA was performed to investigate the effects of pushing method on energetic cost, HR, running speed, and stride length. Sex was included as a fixed factor. A post-hoc Tukey HSD test was performed to compare whether pushing methods differed from each other in their influence of the main variables. All statistical analyses were conducted using SPSS 23.0 (Armonk, NY, USA).

## Results

Male and female participants experienced similar physiological and biomechanical changes during SR conditions as sex did not have a significant effect within any ANOVA model.

Although participants were instructed to maintain their self-selected speed, the addition of a stroller significantly slowed running speed (p = 0.001). Pushing method had varying effects on running speed across participants, with mean speed being highest for Non-SR (3.29 ms^-1^ ± 0.48), followed by 2-Hands (3.09 ms^-1^ ± 0.50), Push/Chase (2.92 ms^-1^ ± 0.45), and 1-Hand (2.88 ms^-1^ ± 0.48) (Table 2).

**Table 2:** Tukey HSD post-hoc tests comparing effect of SR-condition on speed [ms^-1^] and stride length [m].

(I) Condition	(J) Condition	Speed [ms^-1^]		Stride Length [m]	
		Mean Difference (I-J)	Sig.	Mean Difference (I-J)	Sig.
**No Stroller**	1-Hand	.4162	**0.026**	.3073	**.020**
	2-Hands	0.2006	0.503	.2323	.109
	Push/Chase	0.374	**0.054**	.3047	**.022**
**1-Hand**	No Stroller	-.4162	**0.026**	-.3073	**.020**
	2-Hands	-0.2156	0.440	-.0750	.878
	Push/Chase	-0.0422	0.991	-.0027	1.000
**2-Hands**	No Stroller	-0.2006	0.503	-.2323	.109
	1-Hand	0.2156	0.440	.0750	.878
	Push/Chase	0.1734	0.622	.0724	.889
**Push/Chase**	No Stroller	-0.374	**0.054**	-.3047	**.022**
	1-Hand	0.0422	0.991	.0027	1.000
	2-Hands	-0.1734	0.622	-.0724	.889

Although pushing method had a significant effect on running speed, no significant changes in HR (p = 0.393) or energetic cost (p = 0.080) (Table 3) were observed. Changes in lower-limb kinematics were observed, as SR significantly shortened stride length in comparison with Non-SR (p = 0.0003) (Tables 2 and 3).

**Table 3:** Kinematic and energetic values across conditions (Mean ± SD).

	Non-SR		1-Hand		2-Hands		Push/Chase	
	Mean	SD (±)	Mean	SD (±)	Mean	SD (±)	Mean	SD (±)
**Stride Length** [m]	2.40	0.36	2.05*	0.33	2.25	0.30	2.11*	0.31
**Speed** [m*s^-1^]	3.29	0.48	2.88*	0.48	3.09	0.50	2.92*	0.45
**Cost** [Joules*min^-1^]	53.48	10.73	51.50	11.89	53.96	10.48	53.06	11.09
**HR** [bpm]	165.1	17.2	165.1	17.0	170.8	15.1	164.8	18.2

* = sig. diff from Non-SR

### Participant preference

Of the 16 participants, 11 preferred the 2-Hands, 4 preferred Push/Chase, and 1 preferred the 1-Hand SR condition to the other SR conditions.

## Discussion

The purpose of this study was to investigate the effects of SR and how pushing method influences physiological and biomechanical factors. The data indicate a significant decrease in speed when participants ran with a stroller, but no concurrent significant change in energetic cost or HR. As has been shown continuously throughout the study of human locomotion, individuals will protect their rate of energetic burden by changing their behavior, and this is most clearly shown by people decreasing their speed [19–22]. Given the clear decreases in speed shown here, stroller running can be included in the list of ‘challenging’ human locomotor regimes, which also include incline walking and burden carrying [21–23]. These tasks consistently drive down speed, which effectively maintains a constant rate of energy usage by the person, exactly as was shown in the present study. [20,22]

### Pushing methods

The results of the present study indicate that SR speed and stride length differ from independent running and that there are significant differences between pushing methods. The post-hoc Tukey HSD test revealed gradients in speed and stride length changes when comparing Non-SR to SR conditions. Non-SR stride length was significantly longer than stride length during 1-Hand (p = 0.020) and Push/Chase (p = 0.022) SR conditions, with similar patterns observed for speed. However, 2-Hand SR showed no significant differences in speed and stride length from the other SR conditions or Non-SR (Table 2). These results show then that the Push/Chase and 1-Handed SR methods are the most disruptive to running kinematics, while the 2-Hands method results in a speed and stride length most similar to Non-SR. These findings corroborate those of O’Sullivan et al (2015) who observed no significant change in stride length or speed when pushing a stroller with both hands. Unfortunately other studies did not explicitly control for pushing method so it is not clear how their results are influenced by stroller posture. The differences between SR conditions themselves may explain conflicting results between prior studies regarding the effect of SR on lower limb kinematics (Table 1).

### Participant preference

Given that 69% of our participants and 51% of the observed stroller runners on public paths all preferred the 2-Handed SR, it seems this is an SR condition that results in the least perturbation of typical running behavior. The results here show that 2-handed SR is in the middle of a continuum of changes in metabolic cost, speed and stride length. Future work should investigate how pushing method preferences might change as an individual becomes more attuned to running with a stroller, how methods might change over very long distances, and how energetic cost patterns might differ between individuals familiar or unfamiliar with SR.

### Physical activity

Postpartum men and women experience declines in physical and mental health and maintaining physical activity during this period of parenthood is often difficult with the added responsibility of caring for a child [2–3]. The present findings suggest that individuals looking to run with a stroller as a form of physical activity should consider implementing different pushing methods depending on their specific fitness objectives. For example, the 2-Hands method may be optimal for individuals looking to reduce the physiological burden of SR as it showed the smallest kinematic change of all SR conditions. However, the Push/Chase or 1-Hand method may be optimal for those looking to further improve cardiovascular health or physical fitness through SR, given the increased energetic burden.

### Energetic cost model

In order to predict the impact of the different SR conditions on metabolic cost, a linear regression was created for each condition which included mass, speed and energetic cost. The models suggest that when speed is maintained, running with a stroller increases cost between 5–8% depending on the pushing method. As expected, the 2-Hands method is the most economical and the Push/Chase method is the most energetically costly. To predict the cost of a runner of a different mass, please go to https://tinyurl.com/stroller-running-calculator.

## Conclusion

The results from the present study suggest that significant changes to both kinematics and physiology occur when stroller running and that different pushing methods result in graded reductions in running speed and stride length. Implementing different pushing methods may allow individuals to adjust their running routine to meet specific fitness objectives.

## Supporting information

S1 Table Underlying data. (XLSX)
